# Systematic genetic analysis of GSDMD, GSDME, and MLKL in PANoptosis

**DOI:** 10.1016/j.jbc.2026.113370

**Published:** 2026-07-28

**Authors:** Omkar Indari, Prashant Giri, Rebecca E. Tweedell, Thirumala-Devi Kanneganti

**Affiliations:** Department of Immunology, St Jude Children's Research Hospital, Memphis, Tennessee, USA

**Keywords:** PANoptosis, PANoptosome, inflammasome, cell death, innate immunity, executioner, gasdermin, GSDMD, GSDME, MLKL, NLR, NLRP3, NLRP12, NLRC5, ZBP1, AIM2, RIPK1, RIPK3, caspase, caspase-1, caspase-8

## Abstract

Cell death is a key innate immune effector mechanism. While it is beneficial for pathogen clearance, excess lytic cell death is linked to inflammation, pathology, and disease. PANoptosis is an innate immune, lytic, inflammatory cell death pathway initiated by innate immune sensors and driven by caspases and RIPKs, with roles in infection, inflammatory disease, and cancer. During PANoptosis, caspases and RIPKs activate multiple pore-forming “executioner” proteins, including gasdermins and mixed lineage kinase domain-like protein (MLKL), which lead to membrane damage and the release of damage-associated molecular patterns and cytokines. While some have begun to define cell death pathways by their pore-forming executioners, the specific executioner(s) genetically required to drive PANoptosis remain unclear. Therefore, we performed a comprehensive genetic analysis of gasdermin D (GSDMD), gasdermin E (GSDME), and MLKL across triggers that drive PANoptosis. Deletion of GSDMD, GSDME, or MLKL individually did not fully block PANoptosis, suggesting these molecules are compensatory in executing PANoptosis. Furthermore, combined deletion of GSDMD, GSDME, and MLKL generally provided greater protection than any single or double deletion, but 20%–50% of cells still died, suggesting involvement of additional executioners that remain unidentified. Overall, our study suggests that defining cell death pathways purely by executioners is error-prone, as the activation of different pore-forming executioners is variable, context-dependent, and redundant. This further suggests that targeting individual executioners will not be sufficient to block cell death in disease contexts where PANoptosis drives pathology; targeting the upstream molecules, such as innate sensors or PANoptosome complex components, will be needed for therapeutic efficacy in infection, inflammatory disease, and cancer.

The innate immune response is the first line of defense against pathogens and homeostatic disruptions. Innate immune pattern recognition receptors (PRRs) respond to pathogen-associated molecular patterns (PAMPs) and damage-associated molecular patterns (DAMPs) to initiate an immune response, with cell death and cytokine release as key mechanisms (1). Programmed cell death pathways are generally classified as nonlytic and lytic pathways. The nonlytic pathway, apoptosis, is often characterized as non-inflammatory (2). In contrast, lytic forms of cell death, such as pyroptosis, necroptosis, and PANoptosis, are characterized by cell membrane rupture and the release of cellular contents, including cytokines and DAMPs, which contribute to inflammation (3, 4, 5, 6, 7, 8). As such, lytic cell death is crucial in host defense. However, excess cell death is linked to inflammation, pathology, and disease (9, 10). Therefore, balancing the execution of cell death is critical for eliminating infections while preventing inflammatory pathology.

Membrane pore formation, or membrane damage, in lytic cell death is generally driven by specialized pore-forming “executioner” proteins; these include gasdermin D (GSDMD) (11, 12, 13), gasdermin E (GSDME) (14), and mixed lineage kinase domain-like pseudokinase (MLKL) (15, 16). Canonically, cleavage of GSDMD by caspase-1/11 (mouse) or caspase-4 (human) occurs in pyroptosis (11, 12), and activation of MLKL *via* RIPK3-mediated phosphorylation occurs in necroptosis (15, 16). These observations have led some to begin defining these cell death pathways by the specific executioner involved. However, more recent studies have shown that these pore-forming molecules can be activated in other contexts. For instance, GSDMD can be activated by caspase-8 (17, 18), and GSDME can be cleaved by caspase-3 (14) or granzymes (19), thereby forming membrane pores and promoting cell lysis. Furthermore, GSDMD, GSDME, and MLKL can all be activated during PANoptosis, an innate immune, lytic, inflammatory cell death pathway initiated by innate immune sensors and driven by caspases and RIPKs through PANoptosome complexes (5, 6, 7, 20). PANoptosis has been implicated in infectious diseases, inflammatory conditions, and cancers (5, 6, 9, 10, 11, 12, 22, 23, 24). While several molecularly distinct PANoptosomes have been identified to date (5, 6, 21, 22, 23, 24, 25), in all cases, these shared executioners are ultimately activated to drive lytic cell death and the release of inflammatory mediators, such as cytokines and DAMPs (5, 6, 22, 23, 25, 26).

While the phenotypic activation of executioner molecules in lytic cell death has been observed in many contexts, our understanding of the contribution of each of these pore-forming molecules alone and together in driving PANoptosis induced by different triggers is limited. Therefore, in the current study, we used genetic approaches to assess the executioner proteins GSDMD, GSDME, and MLKL across a wide range of infectious and sterile stimuli. We found that these pore-forming molecules have both distinct and overlapping roles in executing PANoptosis. Specifically, deletion of any single executioner could not fully inhibit cell death, regardless of the trigger. Biochemical analysis showed that the deletion of individual executioners did not reduce the activation of upstream caspases or the remaining executioners, suggesting intact upstream signaling and compensatory dynamics among these molecules. Deletion of all three (GSDMD, GSDME, and MLKL) generally provided the highest level of protection from PANoptosis; however, this was still incomplete, suggesting additional executioners remain to be identified. These findings show that defining cell death pathways based on canonical executioner activation is inaccurate, as the executioner molecules are variable, context-dependent, and redundant downstream of the sensor engagement and cell death complex formation. This further improves our understanding of the overall execution of PANoptosis, informing therapeutic strategies to target multiple executioner molecules, or the upstream sensors or essential PANoptosome complex components, and block inflammatory cell death in infection, autoinflammatory diseases, neurodegenerative diseases, and cancer.

## Results

### GSDMD, GSDME, and MLKL contribute to the execution of ZBP1-induced PANoptosis, along with unknown executioners

**Figure 1:**
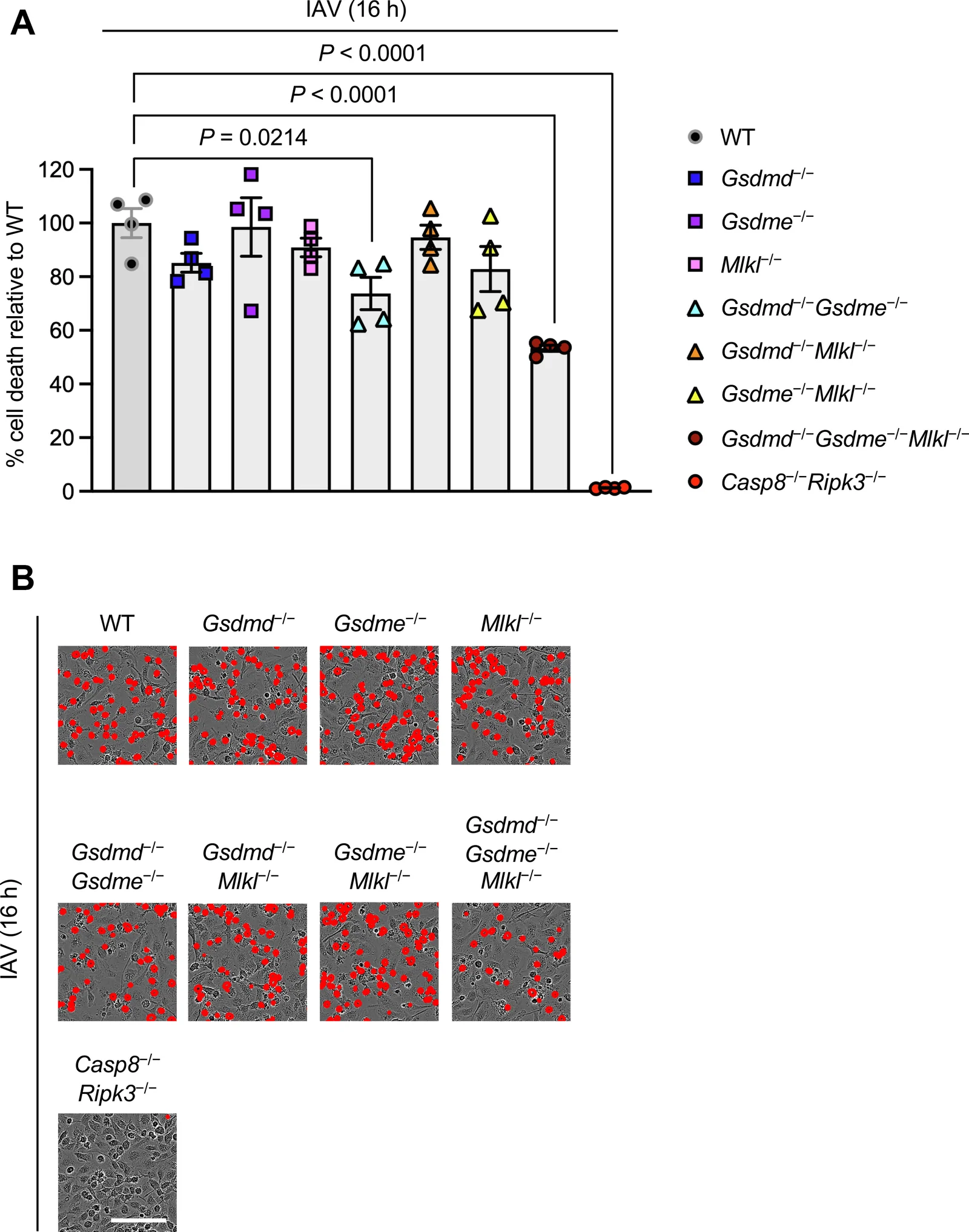
**GSDMD, GSDME, and MLKL contribute to the execution of ZBP1-induced PANoptosis, along with unknown executioners.** Bone marrow-derived macrophages (BMDMs) derived from wild type (WT) and the indicated executioner knockout mice were infected with influenza A virus (IAV; MOI 25) and cell death analysis was performed using IncuCyte live cell imaging. *A*, quantification of the percent of relative cell death (propidium iodide (PI)^+^ cells/well) 16 h after IAV infection, when WT cell death reaches its peak. The relative cell death of individual knockout groups was calculated based on the WT control from that particular experiment. *B*, representative images of cell death at 16 h post-infection, where the *red masks* represent PI^+^ cells undergoing lytic cell death. Scale bar = 100 μm. Data shown in (*A*) are pooled from representative experiments with n = 4 technical replicates from a single well per genotype; data are displayed as mean ± SEM. Similar results were obtained in at least 3 independent biological replicate experiments. Statistical comparisons were made using one-way ANOVA with Dunnett’s multiple comparisons test (*A*), and only statistically significant comparisons are annotated in the figure.

Influenza A virus (IAV) is sensed by ZBP1 to form the ZBP1-PANoptosome and drive PANoptosis (5, 20, 27, 28). While one study suggested a role for MLKL in driving IAV-induced PANoptosis (29), follow-up studies demonstrated that MLKL is not required to execute this cell death (26). Yet, the involvement of other executioners in driving IAV-induced PANoptosis is not known. To test this, we used bone marrow-derived macrophages (BMDMs) infected with IAV as a model system. Wild type (WT), *Gsdmd*^−/−^, *Gsdme*^−/−^, and *Mlkl*^−/−^ BMDMs were used to test the role of individual executioners. Propidium iodide (PI) uptake was utilized as a readout of cell death, as it reflects the loss of membrane integrity and indicates cell death in response to PANoptotic triggers (25, 30, 31). To characterize the kinetics of IAV-induced cell death, we performed time-course analysis of cell death in WT and executioner-deficient BMDMs (Fig. S1). Based on these analyses, we selected the 16 h time point for our comparative analyses, as cell death reached near-maximal levels in WT cells at this time. As previously shown (26), the loss of MLKL alone did not protect against ZBP1-mediated PANoptosis in response to IAV (Fig. 1). Moreover, we found that the loss of any single executioner did not significantly protect against IAV-mediated cell death (Fig. 1). These results suggest that each executioner can be compensated for by another executioner to drive IAV-induced lytic cell death.

To further test this functional redundancy among individual executioners in driving IAV-induced lytic cell death, we leveraged BMDMs from combinational executioner double knockout (DKO; *Gsdmd*^−/−^*Gsdme*^−/−^, *Gsdmd*^−/−^*Mlkl*^−/−^, and *Gsdme*^−/−^*Mlkl*^−/−^) and triple knockout (TKO; *Gsdmd*^−/−^*Gsdme*^−/−^*Mlkl*^−/−^) mice. We observed partial protection against cell death in *Gsdmd*^−/−^*Gsdme*^−/−^ BMDMs (∼25%) but not in the other DKOs (Fig. 1). When all three executioners were absent (*Gsdmd*^−/−^*Gsdme*^−/−^*Mlkl*^−/−^), we observed an even higher level of protection (∼50%, *p* < 0.0001) from IAV-induced lytic cell death (Fig. 1). As a control, we used BMDMs lacking core PANoptosome components, caspase-8 and RIPK3 (*Casp8*^−/−^*Ripk3*^−/−^ DKO). As previously reported (5, 26), *Casp8*^−/−^*Ripk3*^−/−^ BMDMs were fully protected from IAV-induced PANoptosis (Fig. 1). Given that deletion of all three executioners (*Gsdmd*^−/−^*Gsdme*^−/−^*Mlkl*^−/−^) did not reach the level of protection observed with deletion of core PANoptosome components (*Casp8*^−/−^*Ripk3*^−/−^), our findings suggest the involvement of additional executioner proteins in conjunction with GSDMD, GSDME, and MLKL that drive IAV-induced PANoptosis.

### GSDMD partially drives AIM2*-*induced PANoptosis, with additional contributions from unknown executioners

**Figure 2:**
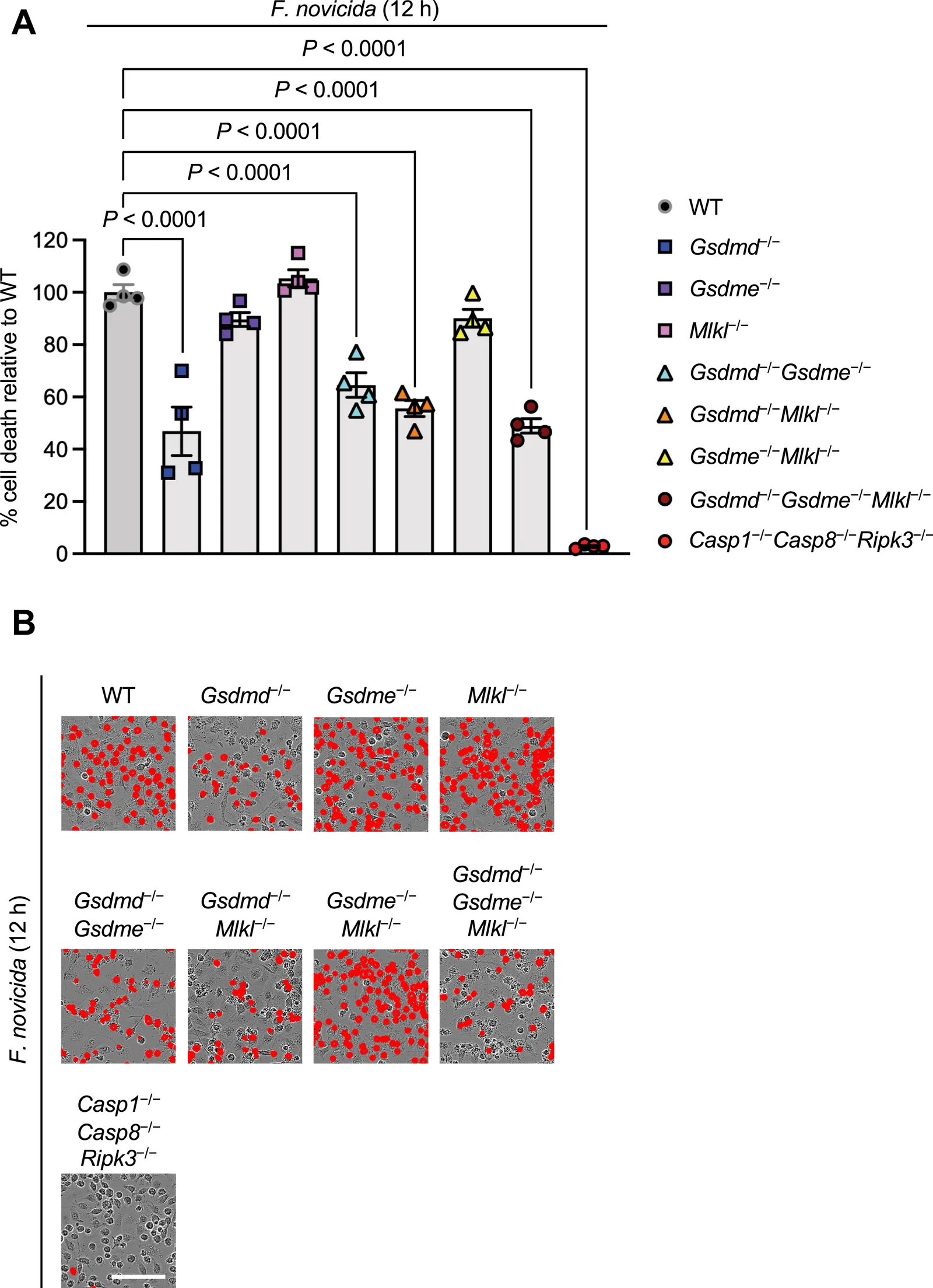
**GSDMD partially drives AIM2*-*induced PANoptosis, with additional contributions fromunknown executioners.** Bone marrow-derived macrophages (BMDMs) derived from wild type (WT) and the indicated executioner knockout mice were infected with *F*. *novicida* (MOI 100) and cell death analysis was performed using IncuCyte live cell imaging. *A*, quantification of the percent of relative cell death (propidium iodide (PI)^+^ cells/well) 12 h after *F*. *novicida* infection, when cell death in WT reaches its peak. The relative cell death of individual knockout groups was calculated based on the WT control from that particular experiment. *B*, representative cell death images at 12 h post-infection, where the *red masks* represent PI^+^ cells undergoing lytic cell death. Scale bar = 100 μm. Data shown in (*A*) are pooled from representative experiments with n = 4 technical replicates from a single well per genotype; data are displayed as mean ± SEM. Similar results were obtained in at least 3 independent biological replicate experiments. Statistical comparisons were made using one-way ANOVA with Dunnett’s multiple comparisons test (*A*), and only statistically significant comparisons are annotated in the figure.

We next sought to test the role of executioners in driving PANoptosis in response to a trigger that signals through a different PANoptosome. Therefore, we utilized a *Francisella novicida* infection model wherein PANoptosis is driven through the AIM2-PANoptosome (21). We temporally assessed cell death during *F*. *novicida* infection (Fig. S2) and selected the timepoint at which cell death plateaued in WT cells for endpoint comparisons (Fig. 2). A previous study suggested a role for GSDMD in driving *F*. *novicida* infection-induced lytic cell death (32); however, the role of other executioner proteins has yet to be tested. We observed that *Gsdmd*^−/−^ BMDMs showed partial, but significant, protection (∼50%, *p* < 0.0001) against *F*. *novicida*-induced PANoptosis compared to WT cells (Fig. 2), as previously suggested (32). However, this protection was not complete, suggesting multiple executioners are involved. No protection against cell death was observed in *Gsdme*^−/−^ or *Mlkl*^−/−^ BMDMs (Fig. 2).

Given the redundancy that we observed among the three executioner proteins in IAV-induced PANoptosis (Fig. 1), we assessed whether this also occurred in response to *F*. *novicida*. We found that any DKOs and the TKO lacking GSDMD were similar to *Gsdmd*^−/−^ BMDMs and showed partial but significant protection from cell death (Fig. 2). As a positive control, we infected BMDMs lacking key PANoptosome components, caspase-1, caspase-8, and RIPK3 (*Casp1*^−/−^*Casp8*^−/−^*Ripk3*^−/−^ TKO mice) with *F*. *novicida*. We found that *Casp1*^−/−^*Casp8*^−/−^*Ripk3*^−/−^ BMDMs were completely protected against *F*. *novicida*-induced lytic cell death (Fig. 2). Altogether, these data suggest that GSDMD contributes to PANoptosis driven by *F*. *novicida*, and that additional yet-to-be-identified executioner molecule(s) also cooperate to execute this cell death.

### GSDME partially drives NLRC5/NLRP12*-*induced PANoptosis, with additional contributions from GSDMD, MLKL, and unknown executioners

Having found redundant roles for executioner molecules in driving PANoptosis, we next sought to extend our study to PANoptosis triggered by other inflammatory conditions. The DAMP heme is released under hemolytic conditions and can synergize with PAMPs or the cytokine TNF to trigger PANoptosis (22, 23). These combinations induce PANoptosis *via* the NLRC5- and NLRP12-PANoptosome (22, 23). In this context, GSDME has been shown to have a partial role in executing cell death (23), but the role of other executioners in this model remains to be investigated.

**Figure 3:**
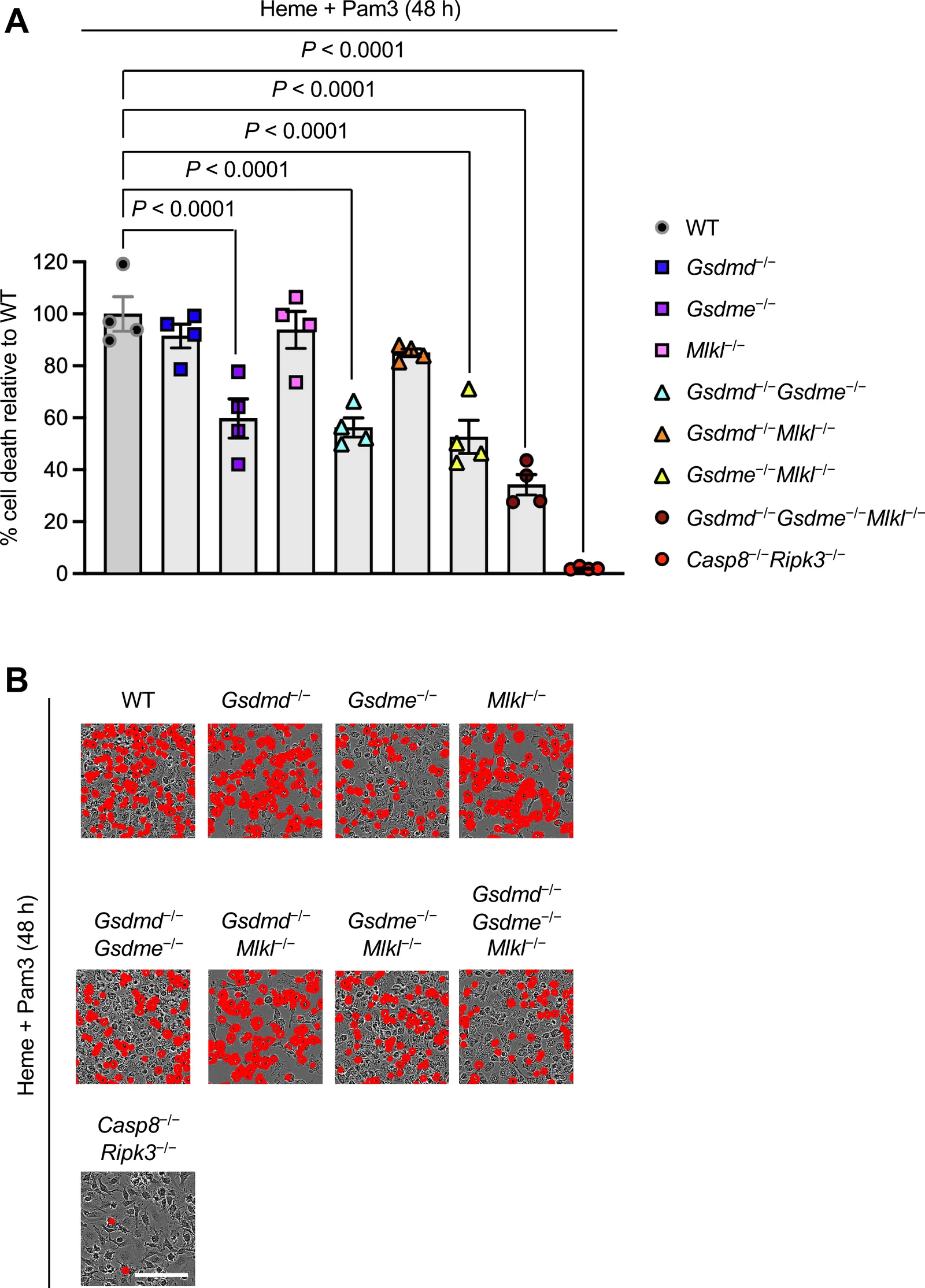
**GSDME partially drives NLRC5/NLRP12*-*induced PANoptosis, with additional contributions from GSDMD, MLKL, and unknown executioners.** Bone marrow-derived macrophages (BMDMs) derived from wild type (WT) and the indicated executioner knockout mice were stimulated with heme plus Pam3 and cell death analysis was performed using IncuCyte live cell imaging. *A*, quantification of the percent of relative cell death (propidium iodide (PI)^+^ cells/well) 48 h after heme plus Pam3 stimulation, when cell death in WT reaches its peak. The relative death of individual knockout groups was calculated based on the WT control from that particular experiment. *B*, representative cell death images at 48 h post-stimulation, where the *red masks* represent PI^+^ cells undergoing lytic cell death. Scale bar = 100 μm. Data shown in (*A*) are pooled from representative experiments with n = 4 technical replicates from a single well per genotype; data are displayed as mean ± SEM. Similar results were obtained in at least 3 independent biological replicate experiments. Statistical comparisons were made using one-way ANOVA with Dunnett’s multiple comparisons test (*A*), and only statistically significant comparisons are annotated in the figure.

As previously reported (23), we found that *Gsdme*^−/−^ BMDMs exhibited a significant reduction in cell death compared to WT controls upon induction of PANoptosis by heme plus Pam3, providing ∼40% protection (*p* < 0.0001; Fig. 3). BMDMs lacking other individual executioners, *Gsdmd*^−/−^ and *Mlkl*^−/−^, showed a level of cell death similar to that of WT cells (Figs. 3 and S3). To assess the combined roles of the executioners, we leveraged executioner DKO and TKO BMDMs. Similar to *Gsdme*^−/−^ BMDMs, DKO cells lacking GSDME (*i.e.*, *Gsdmd*^−/−^*Gsdme*^−/−^ and *Gsdme*^−/−^*Mlkl*^−/−^) provided ∼40% protection against cell death induced by heme plus Pam3. Meanwhile, *Gsdmd*^−/−^*Mlkl*^−/−^ cells did not show any protection against cell death. Furthermore, *Gsdmd*^−/−^*Gsdme*^−/−^*Mlkl*^−/−^ TKO BMDMs showed a numerically higher level of protection compared to any single KO or DKO group, wherein cell death was reduced by ∼65% (*p* < 0.0001; Fig. 3), highlighting that GSDME function can be partially compensated for by GSDMD and MLKL together. *Casp8*^−/−^*Ripk3*^−/−^ BMDMs lacking vital PANoptosome components were completely protected against lytic cell death induced by heme plus Pam3 (Fig. 3) as observed previously (22, 23). These findings suggest involvement of other unknown executioners regulated by these PANoptosis components that contribute to the residual cell death observed in *Gsdmd*^−/−^*Gsdme*^−/−^*Mlkl*^−/−^ TKO cells.

### GSDME partially drives cytokine-induced PANoptosis, with additional contributions from GSDMD, MLKL, and unknown executioners

**Figure 4:**
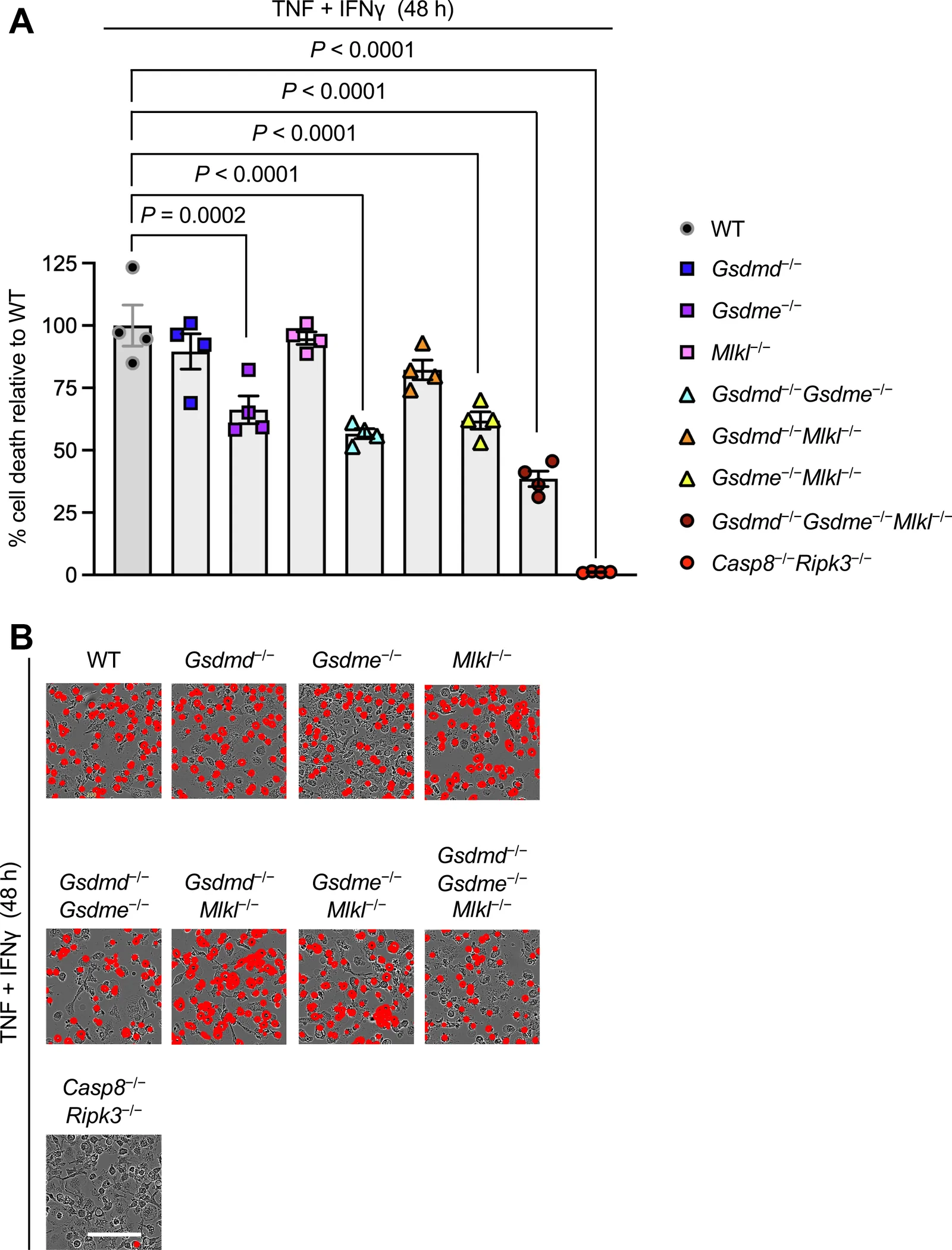
**GSDME partially drives cytokine-induced PANoptosis, with additional contributions from GSDMD, MLKL, and unknown executioners.** Bone marrow-derived macrophages (BMDMs) derived from wild type (WT) and the indicated executioner knockout mice were stimulated with TNF plus IFNγ and cell death analysis was performed using IncuCyte live cell imaging. *A*, quantification of the percent of relative cell death (propidium iodide (PI)^+^ cells/well) 48 h after TNF plus IFNγ stimulation, when cell death in WT reaches its peak. The relative death of individual knockout groups was calculated based on the WT control from that particular experiment. *B*, representative cell death images at 48 h post-stimulation, where the *red masks* represent PI^+^ cells undergoing lytic cell death. Scale bar = 100 μm. Data shown in (*A*) are pooled from representative experiments with n = 4 technical replicates from a single well per genotype; data are displayed as mean ± SEM. Similar results were obtained in at least 3 independent biological replicate experiments. Statistical comparisons were made using one-way ANOVA with Dunnett’s multiple comparisons test (*A*), and only statistically significant comparisons are annotated in the figure.

After observing consistent compensatory roles among GSDMD, GSDME, and MLKL in driving PANoptosis, we continued to investigate whether this phenomenon occurs in other PANoptosis settings. Combined stimulation with the cytokines TNF and IFNγ induces PANoptosis, with a known contribution of GSDME to cell death in this context (6). Yet, the functional relationship among GSDMD, GSDME, and MLKL in executing cell death in this context has not been studied. We found that TNF plus IFNγ stimulation produced cell death patterns in our executioner KOs (Figs. 4 and S4) that were similar to those observed with heme plus Pam3 treatment (Figs. 3 and S3). Specifically, *Gsdme*^−/−^, *Gsdmd*^−/−^*Gsdme*^−/−^, and *Gsdme*^−/−^*Mlkl*^−/−^ BMDMs exhibited significant protection (∼40% reduction in cell death, *p* < 0.0001) compared to WT BMDMs (Fig. 4). A partial reduction in cell death was observed in *Gsdmd*^−/−^*Mlkl*^−/−^ BMDMs, though this was not statistically significant, and the loss of *Gsdmd* or *Mlkl* in isolation did not show any protection (Fig. 4). As with other PANoptosis triggers, executioner TKO (*Gsdmd*^−/−^*Gsdme*^−/−^*Mlkl*^−/−^) BMDMs showed the highest level of protection (∼60%, *p* < 0.0001) compared to WT cells (Fig. 4). In particular, *Gsdme*^−/−^ modestly affected the onset of cell death, while *Gsdmd*^−/−^*Gsdme*^−/−^*Mlkl*^−/−^ TKO BMDMs showed comparatively delayed and a reduced level of cell death overall (Fig. S4). Similar to previous observations (6), *Casp8*^−/−^*Ripk3*^−/−^ BMDMs were fully protected from TNF plus IFNγ-induced PANoptosis (Fig. 4). These findings suggest a role for GSDME in driving cell death induced by TNF plus IFNγ that can be partially compensated for by both GSDMD and MLKL, with additional executioners still driving the cell death when GSDMD, GSDME, and MLKL are deleted.

### GSDMD, GSDME, and MLKL contribute to the execution of RIPK1-induced PANoptosis, along with unknown executioners

**Figure 5:**
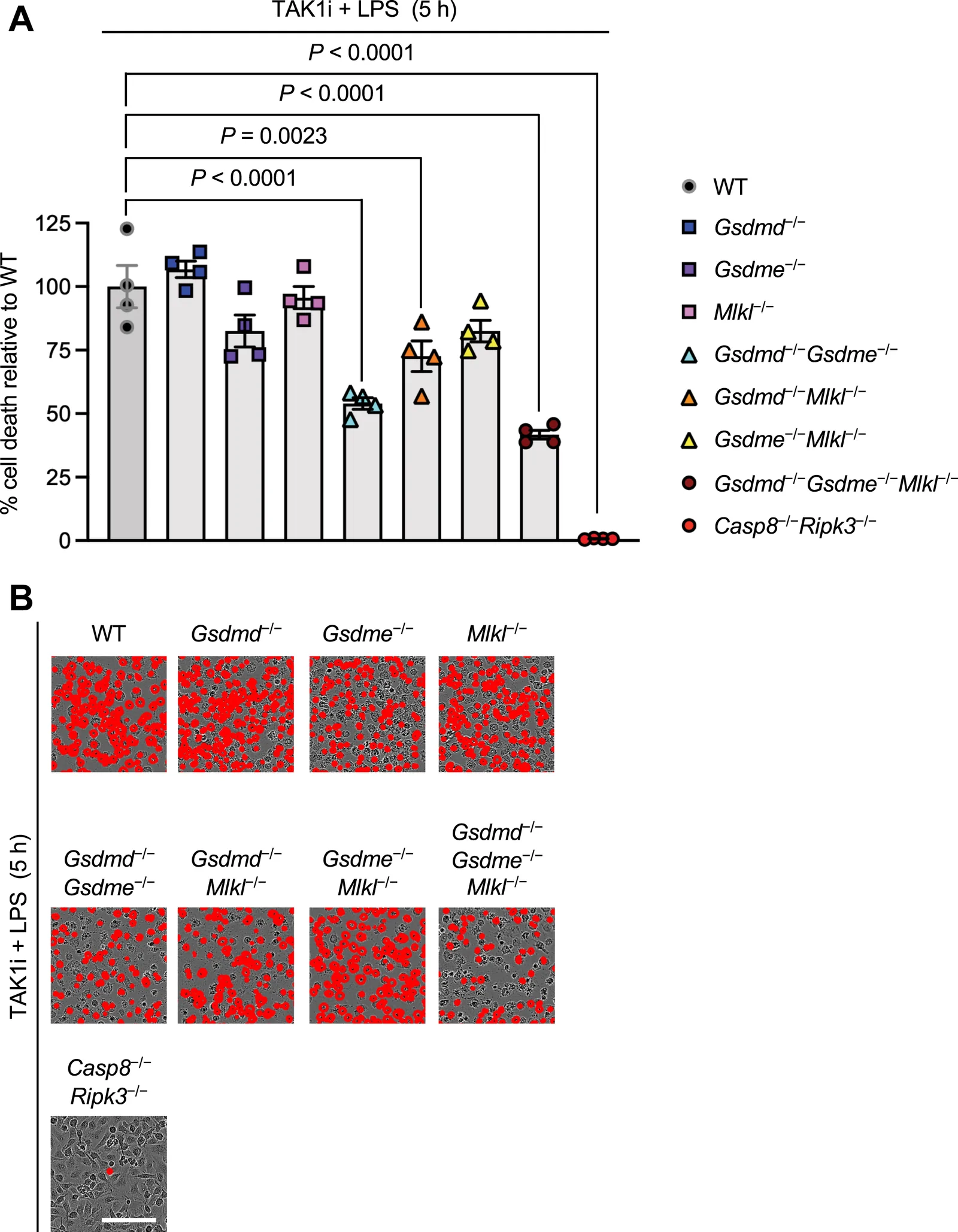
**GSDMD, GSDME, and MLKL contribute to the execution of RIPK1-induced PANoptosis, along with unknown executioners.** Bone marrow-derived macrophages (BMDMs) derived from wild type (WT) and the indicated executioner knockout mice were stimulated with TAK1 inhibitor (TAK1i) plus LPS and cell death analysis was performed using IncuCyte live cell imaging. *A*, quantification of the percent of relative cell death (propidium iodide (PI)^+^ cells/well) 5 h after TAK1i plus LPS stimulation, when cell death in WT reaches its peak. The relative death of individual knockout groups was calculated based on the WT control from that particular experiment. *B*, representative cell death images at 5 h post-stimulation, where the *red masks* represent PI^+^ positive cells undergoing lytic cell death. Scale bar = 100 μm. Data shown in (*A*) are pooled from representative experiments with n = 4 technical replicates from a single well per genotype; data are displayed as mean ± SEM. Similar results were obtained in at least 3 independent biological replicate experiments. Statistical comparisons were made using one-way ANOVA with Dunnett’s multiple comparisons test (*A*), and only statistically significant comparisons are annotated in the figure.

Finally, we investigated the role of executioner molecules in driving RIPK1-mediated PANoptosis triggered by the combination of TAK1 inhibition (TAK1i) and the PAMP, lipopolysaccharide (LPS). TAK1 inhibition causes TNF signaling and the induction of RIPK1-mediated PANoptosis when combined with LPS (33, 34). Although previous studies showed the activation of GSDMD and MLKL in TAK1i plus LPS-induced PANoptosis (33, 34), the role of GSDME and potential compensation among executioner molecules in this context remains unknown. Cells lacking *Gsdmd*, *Gsdme*, or *Mlkl* alone showed comparable cell death to WT BMDMs at 5 h post-stimulation (Figs. 5 and S5), suggesting that no single executioner drives cell death induced by TAK1i plus LPS. We observed that GSDMD DKO BMDMs (*i*.*e*., *Gsdmd*^−/−^*Gsdme*^−/−^ and *Gsdmd*^−/−^*Mlkl*^−/−^) showed significant, though partial, protection from PANoptosis induced by TAK1i plus LPS (Fig. 5). Moreover, cells lacking all three executioners (*Gsdmd*^−/−^*Gsdme*^−/−^*Mlkl*^−/−^) demonstrated the highest level of protection compared to any single or double KOs (∼55%, *p* < 0.0001, Fig. 5). These data highlight that executioner molecules may compensate for one another in mediating TAK1i plus LPS-induced PANoptosis, but that additional executioners are also involved. As reported previously (33, 34), *Casp8*^−/−^*Ripk3*^−/−^ cells were completely rescued from TAK1i plus LPS-driven PANoptosis (Fig. 5). Consistent with our previous findings (Figure 1, Figure 2, Figure 3, Figure 4), the residual cell death observed in *Gsdmd*^−/−^*Gsdme*^−/−^*Mlkl*^−/−^ BMDMs suggests the involvement of other molecule(s) in executing TAK1i plus LPS-induced lytic cell death.

### Executioners are downstream cell death effectors that do not impact upstream caspase activation or activation of other executioners

To understand the effect that loss of executioners has on the overall biochemical activation of key PANoptosis effector molecules, we performed time-resolved immunoblot analyses. Specifically, we examined activation of caspase-1, caspase-3, caspase-7, and caspase-8 alongside cleavage of GSDMD and GSDME and phosphorylation of MLKL in WT and executioner-deficient BMDMs (*Gsdmd*^−/−^, *Gsdme*^−/−^, and *Mlkl*^−/−^) across three representative cell death stimuli: IAV infection, TAK1i plus LPS, and TNF plus IFNγ.

**Figure 6:**
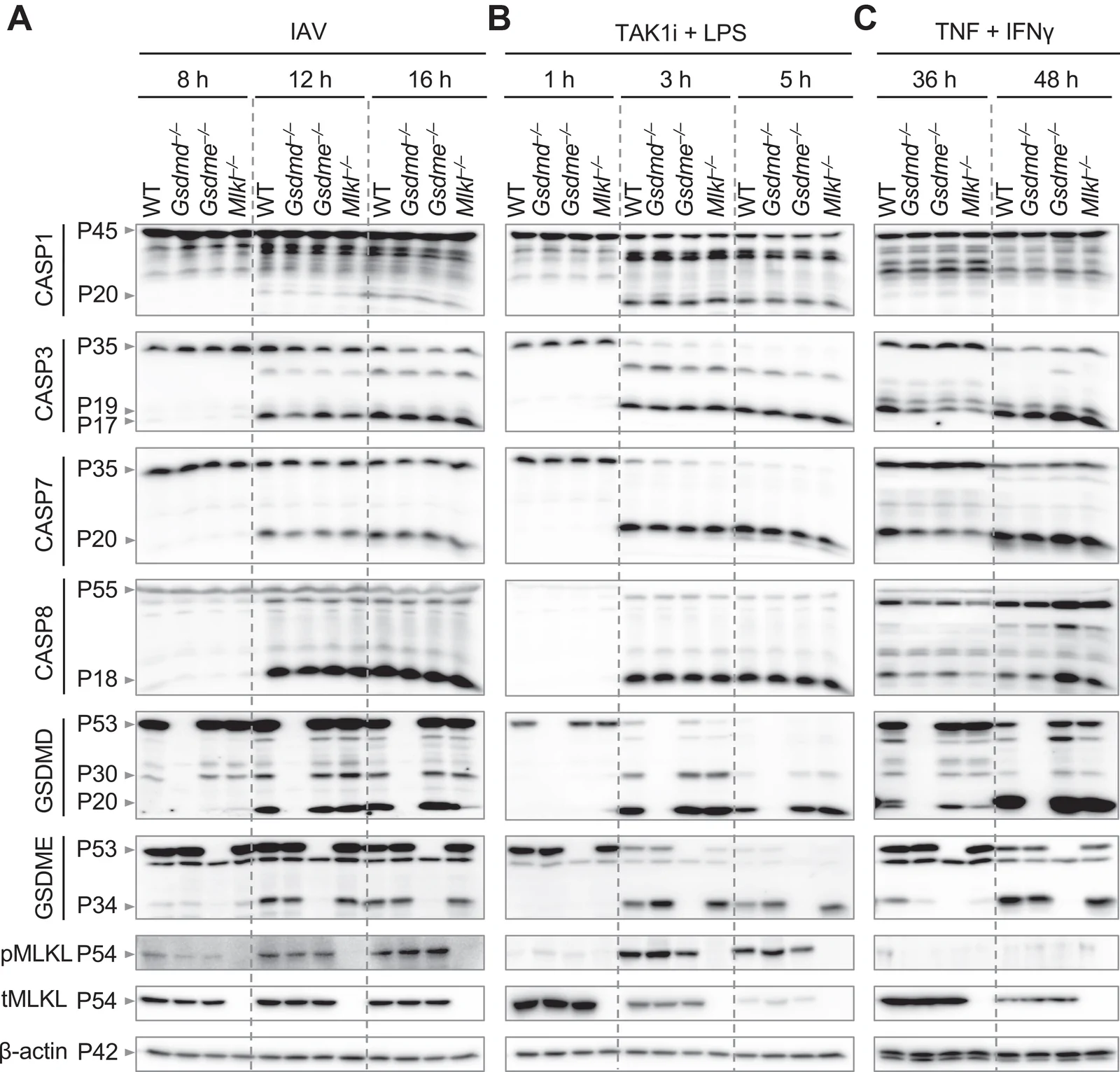
**Caspase and executioner activation kinetics during PANoptosis are not inhibited by executioner deletion.** Wild type (WT), *Gsdmd*^−/−^, *Gsdme*^−/−^, and *Mlkl*^−/−^ bone marrow-derived macrophages (BMDMs) were subjected to (*A*) influenza A virus (IAV) infection, (*B*) TAK1 inhibitor (TAK1i) plus LPS treatment, or (*C*) TNF plus IFNγ treatment. Time-resolved immunoblot analyses of pro- (P45) and activated (P20) caspase-1 (CASP1); pro- (P35) and cleaved (P19/P17) caspase-3 (CASP3); pro- (P35) and cleaved (P20) caspase-7 (CASP7); pro- (P55) and cleaved (P18) caspase-8 (CASP8); pro- (P53), activated (P30), and inactivated (P20) gasdermin D (GSDMD); pro- (P53) and cleaved (P34) gasdermin E (GSDME); phosphorylated MLKL (pMLKL); and total MLKL (tMLKL) are shown. β-actin was used as a loading control. Representative immunoblots from one of at least three independent biological replicate experiments are shown. Similar results were obtained in each experiment.

In WT macrophages, we observed temporal activation of PANoptosis effector molecules including caspases, gasdermins, and/or MLKL, in response to each trigger, as expected (Fig. 6). Deletion of individual executioners did not reduce the activation of upstream caspases (Fig. 6). These findings indicate that upstream cell death signaling remains intact in response to PANoptotic triggers, despite loss of specific executioner proteins.

Given our genetic data suggesting that there is redundancy among executioners during PANoptosis (Figure 1, Figure 2, Figure 3, Figure 4, Figure 5), we next assessed the activation of each executioner when one was deleted. Indeed, we observed activation of other executioners in each individual knockout (Fig. 6). These results suggest that loss of one executioner does not impact the ability of cells to activate other executioners, allowing for functional redundancy in the execution of cell death.

Taken together, these data demonstrate that activation of both upstream signaling components and downstream executioners persists in the absence of individual executioners.

## Discussion

Cell death is central in the innate immune response; therefore, programmed cell death pathways are critically involved in disease progression and represent potentially valuable therapeutic targets (7, 8, 35). Thus, understanding the key molecules that execute PANoptosis is crucial to inform therapeutic strategies. In this study, we systematically and comprehensively investigated the requirement for the executioners GSDMD, GSDME, and MLKL at the genetic level in five distinct, physiologically relevant PANoptotic conditions, each driven by different triggers and involving distinct innate immune sensors. Our findings define stimulus-dependent roles and compensatory functions among executioner proteins in PANoptosis. In response to some triggers, the loss of a single executioner protein can provide some protection against cell death (*e*.*g*., GSDMD in *F*. *novicida*-driven PANoptosis). In response to other triggers, we observed protection against cell death only after two or more executioners were deleted (*e*.*g*., GSDMD and GSDME in PANoptosis driven by TAK1i plus LPS). Despite these trigger-dependent differences, we generally observed the greatest level of protection from PANoptosis in *Gsdmd*^−/−^*Gsdme*^−/−^*Mlkl*^−/−^ cells, highlighting the functional redundancy among executioner molecules independent of the trigger. The exception was in AIM2-mediated PANoptosis in response to *F. novicida* infection, where *Gsdmd*^−/−^ cells and *Gsdmd*^−/−^*Gsdme*^−/−^*Mlkl*^−/−^ cells showed similar protection.

We also observed that deletion of all three executioners could not completely block PANoptosis. This central finding suggests that additional executioner proteins yet to be identified are involved in executing PANoptosis. Furthermore, the potential contribution of other membrane channels such as pannexin-1 (36) has yet to be investigated in the context of PANoptosis. Future studies to identify other executioner molecules downstream of core PANoptosis components will elucidate the complete array of PANoptosome-activated molecules that drive cell death. Species- and cell type-specific differences may also influence cell death pathway regulation. Therefore, future work to extend these analyses to other cell types and human systems will be informative.

Our biochemical analyses demonstrated that cell death signaling and executioner activation persist in the absence of individual executioners. This suggests that the upstream cell death signaling remains intact and is responsible for the remaining cell death, despite executioner deletion. In the absence of executioners, it remains possible that secondary effects of sustained signaling complex formation or non-programmed cell death pathways can impact the observed outcomes. However, since cell death with the triggers tested here can be genetically blocked by deletion of caspase-8 and RIPK3, with or without caspase-1, this suggests programmed cell death dependent on the PANoptosome complex is occurring. Additionally, whether sublytic pore formation or pore repair occur during PANoptosis, especially during the loss of executioner molecules, remains to be determined. Gasdermin pores can exist in sublytic states (37, 38), and membrane repair pathways (39), including calcium-dependent processes such as ESCRT-mediated repair (40), may counteract pore formation and limit cell death.

Therapeutic targeting of these executioner proteins is actively being pursued. For instance, disulfiram blocks GSDMD pore formation and inhibits subsequent cell death, while also inhibiting ALDH, TLR4 signaling, and NF-κB-dependent priming (41, 42, 43). Disulfiram was originally used clinically for alcohol use disorders, and it is now being repurposed for investigation in preclinical and translational studies aimed at modulating inflammatory cell death (43). MLKL inhibitors, such as necrosulfonamide or its analogs (44, 45) and AMG-47a (46), as well as interventions aimed at modulating GSDME activity are also under investigation (47). Several executioner inhibitors block more than one molecule, such as dimethyl fumarate, which blocks both GSDMD and GSDME (48). Although this limits specificity in executioner targeting, our present study highlights that combinatorial executioner targeting may be beneficial to achieve robust protection against PANoptotic cell death, independent of the trigger. This suggests potential utility for combinatorial targeting of executioners in PANoptosis, where executioner redundancy occurs. However, it may be even more effective to target upstream molecules. Our study shows that the executioner molecules downstream are variable, context-dependent, and redundant outputs of the upstream sensors and cell death complex. Therefore, executioners are not the defining feature of the pathway; the cell death complex is. This suggests that the most effective therapy may be to block the upstream biological unit which actually defines the pathway. This framework can be applied broadly across cell death pathways, where pyroptosis is defined by inflammasome-initiated cell death, necroptosis is necrosome-initiated cell death, and PANoptosis is PANoptosome-initiated cell death. Defining cell death pathways based on their upstream sensors and complex formation can provide better insights for therapeutic translation.

Overall, our study provides a comprehensive characterization of the role of the executioner molecules GSDMD, GSDME, and MLKL in driving PANoptosis, highlighting that combined deletion of all three executioners consistently did not fully block PANoptosis, suggesting that additional executioners can still drive this cell death. These findings show that cell death cannot be accurately defined by executioner engagement and will inform the development of therapies for infectious and inflammatory diseases in which PANoptosis plays a central role. As cell death-targeted treatments are a growing area of therapeutic development, the translational potential of targeting these molecules supports future efforts to identify additional PANoptosis regulators.

## Experimental procedures

### Mice

Wild type (WT) C57BL/6J, *Gsdmd*^−/−^ (49), *Gsdme*^−/−^ (50), *Mlkl*^−/−^ (51), *Gsdmd*^−/−^*Gsdme*^−/−^ (52), *Gsdmd*^−/−^*Mlkl*^−/−^ (20), *Gsdmd*^−/−^*Gsdme*^−/−^*Mlkl*^−/−^ (6), *Casp8*^−/−^*Ripk3*^−/−^ (53), and *Casp1*^−/−^*Casp8*^−/−^*Ripk3*^−/−^ (23) mice used in this study have been described previously. *Gsdme*^−/−^*Mlkl*^−/−^ mice were generated in-house by crossing *Gsdme*^−/−^ and *Mlkl*^−/−^ animals. All mice were generated on or extensively backcrossed to the C57BL/6J background. Mice were bred at the Animal Resources Center at St Jude Children's Research Hospital and maintained under specific pathogen-free conditions. Age- and sex-matched male and female 4- to 12-week-old mice were used in this study. Animal studies were conducted under protocols approved by the St Jude Children’s Research Hospital committee on the Use and Care of Animals.

### Mouse bone marrow-derived macrophage differentiation and culture

Primary mouse bone marrow-derived macrophages (BMDMs) were generated through a 6-day differentiation cycle from bone marrow of WT and the indicated mutant mice. The choice of the genetic negative control for each experiment was determined by the known signaling requirements of each stimulus and was intentionally selected to demonstrate maximal protection from cell death. Bones were harvested from mice and flushed to collect bone marrow on day 0 under aseptic conditions. The bone marrow was cultivated in 150 mm dishes with 20 ml of BMDM media composed of Iscove’s Modified Dulbecco’s Medium (Thermo Fisher Scientific, 12440-053) containing 30% L929-conditioned medium, 10% heat-inactivated fetal bovine serum (HI-FBS; Biowest, S1620), 1% non-essential amino acids (Thermo Fisher Scientific, 11140-050), and 1% antibiotic solution of penicillin and streptomycin (Pen-Strep; Thermo Fisher Scientific, 15070-063). Cells were incubated in a humidified incubator containing 5% CO_2_ at 37 °C. An additional 5 ml of BMDM media was added on day 3 and day 5. On day 6, the cells were scraped in PBS, resuspended in BMDM media, counted, and seeded in 24-well plates at a density of 5 × 10^5^ cells/well or in 12-well plates at a density of 1 × 10^6^ cells/well. On the next day, the cells were used for further stimulation with cell death triggers.

### Virus and bacteria culture

The influenza A virus strain A/Puerto Rico/8/34, H1N1 [PR8] (referred as IAV in the manuscript) was produced using 11-day-old embryonated chicken eggs as previously described (54). Briefly, IAV was inoculated into the eggs *via* allantoic inoculation. Viral infection was stopped after 48 h by transferring eggs to 4 °C overnight. The clear fluid was harvested the next day and centrifuged at 4000*g* for 30 min to remove debris. The resulting clear suspension was collected and aliquoted, then stored at −80 °C for further use. The IAV titer was measured by plaque assay in Madin–Darby canine kidney cells.

For bacterial infection, *F*. *novicida* were cultivated in tryptic soy broth containing 0.2% cysteine overnight. The next day, the bacteria were subcultured at 1:10. The bacterial density was measured after 4 to 6 h and then utilized for infection.

### Stimulations and cell death analysis

Cultured BMDMs were first washed with PBS and then treated with specific triggers in media containing 500 ng/ml propidium iodide (PI; Life Technologies, P3566). For IAV infection, BMDMs were infected with IAV (multiplicity of infection (MOI) 25) in Dulbecco’s Modified Eagle Medium (DMEM) with 1% Pen-Strep without HI-FBS. After 1 h, 10% HI-FBS was added. For *F*. *novicida* infection, BMDMs were infected with *F*. *novicida* (MOI 100) in DMEM with 10% HI-FBS without Pen-Strep. After 4 h, the cells were washed with PBS three times, and then DMEM with 10% HI-FBS and 50 μg/ml gentamicin was added. For ligand stimulation, cells were treated with the following in 250 μl DMEM with 10% HI-FBS and 1% Pen-Strep: 5Z-7-oxozeaenol (TAK1 inhibitor, TAK1i; 0.1 μM) plus lipopolysaccharide (LPS; 100 ng/ml), heme (50 μM) plus Pam3CSK4 (Pam3; 500 ng/ml), and TNF (50 ng/ml) plus IFNγ (100 ng/ml). The stimulated cells were cultured at 37 °C and 5% CO_2_ in an IncuCyte S3 or SX5 live-cell automated imaging system (Sartorius) for cell death analysis. The entry of PI into the cell through altered cell permeability was used as an indication of lytic cell death. Cell death was tracked by the acquisition of phase-contrast and fluorescence images (four image fields/well) at the indicated times to monitor the cell death using a 20 × objective. PI^+^ dead cells were marked with a red mask for visualization. Images were analyzed with IncuCyte software. In some cases, a single WT was used to compare against multiple knockout lines inoculated and stimulated at the same time in the same experiment. The data are plotted separately in the figures for optimal visualization; therefore, the same WT cell death curve appears in multiple panels.

### Immunoblot analysis

After appropriate treatments, cells were lysed at the indicated timepoints along with culture supernatants in caspase lysis buffer (containing 10% NP-40, 25 mM DTT and 1 × protease and phosphatase inhibitors (Roche, 11697498001 and 04906837001, respectively)) and 4 × SDS sample loading buffer (with 2-mercaptoethanol) for probing caspase activation. For immunoblot assessment of signaling activation, culture supernatants were removed, cells were washed once with 1 × Dulbecco’s PBS (DPBS; Thermo Fisher Scientific, 14190-250) and lysed in RIPA buffer and 4 × SDS sample loading buffer. All samples were boiled at 100 °C for 10 min before being loaded onto gels. Proteins were resolved on 8 to 12% polyacrylamide gels and transferred onto PVDF membranes (Millipore, IPVH00010) using the Trans-Blot Turbo system. After blocking non-specific binding with 5% skim milk, membranes were incubated overnight with the following primary antibodies against: caspase-1 (AdipoGen, AG-20B-0042, 1:1000), caspase-3 (Cell Signaling Technology [CST], #9662, 1:1000), cleaved caspase-3 (CST, #9661, 1:1000), caspase-7 (CST, #9492, 1:1000), cleaved caspase-7 (CST, #9491, 1:1000), caspase-8 (CST, #4927, 1:1000), cleaved caspase-8 (CST, #8592, 1:1000), GSDMD (Abcam, ab209845, 1:1000), GSDME (Abcam, ab215191, 1:1000), p-MLKL (CST, #37333, 1:1000), t-MLKL (Abgent, AP14272B, 1:1000), and β-actin (Santa Cruz, sc-47778 HRP, 1:5000). All antibodies used in this study were initially validated for specificity using the corresponding knockout cells, where available, prior to their use in downstream experiments. Membranes were then washed and probed with the appropriate horseradish peroxidase (HRP)-conjugated secondary antibodies (Jackson ImmunoResearch Laboratories, anti-mouse [315-035-047] and anti-rabbit [111-035-047], 1:5000). Immunoblot images were acquired on an Amersham Imager using Immobilon Forte Western HRP Substrate (Millipore, WBLUF0500) or SuperSignal West Femto Maximum Sensitivity Substrate (Thermo Fisher Scientific, 34096).

### Statistical analysis

For data analysis and interpretation, GraphPad Prism software (v10, GraphPad Software, Inc) was utilized. For bar graphs, mean percent cell death was calculated from four acquired images per genotype in each experiment. Then data were normalized within each experiment relative to WT, where the WT level of cell death was set to 100%. Relative cell death was calculated according to the formula: Relative cell death (% of WT) = (mean percent cell death in the indicated genotype ÷ mean percent cell death of WT controls from the same experiment) × 100. Normalized data from multiple independent experiments were combined, and data were plotted and shown as the mean ± SEM. Data from representative experiments have been presented. All data are representative of at least three independent experiments. For statistical comparison, each genetic knockout group was compared to the WT control group. Statistical significance for cell death analysis was calculated using one-way ANOVA with Dunnett’s multiple comparisons test. *p* < 0.05 was considered statistically significant.

## Data availability

All data in this study are included in this article. They are available from the corresponding author upon reasonable request.

## Supporting information

This article contains supporting information.

## Conflict of interest

The authors declare that they have no conflicts of interest with the contents of this article.
