# Supplementary material for: Systematic genetic analysis of GSDMD, GSDME, and MLKL in PANoptosis
Source: J Biol Chem. 2026 Jul 28;302(9):113370. doi: 10.1016/j.jbc.2026.113370 (PMC13573193; doi:10.1016/j.jbc.2026.113370)
Supplement: Supplementary file 2 [file mmc2.pdf]

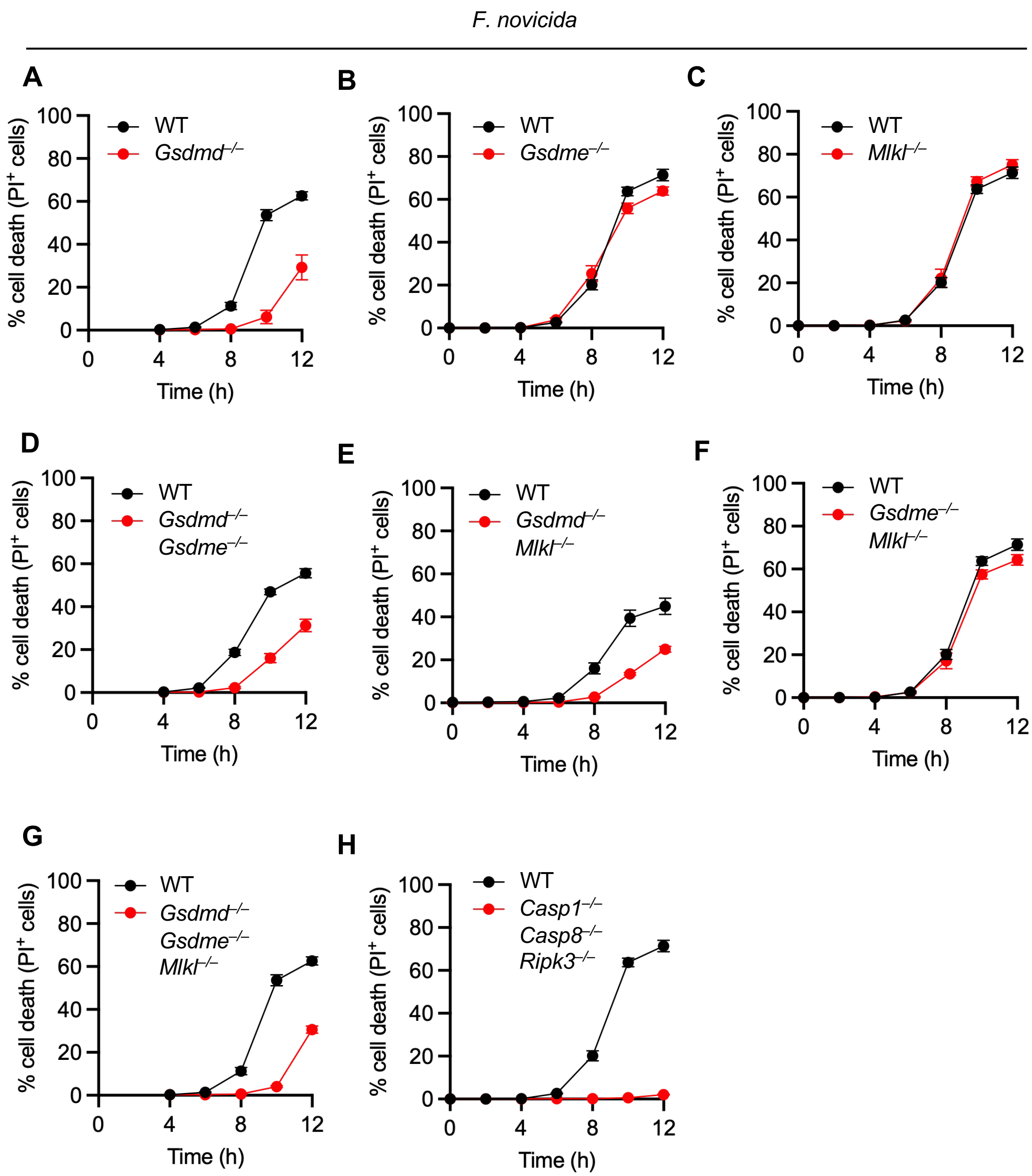

Figure S2: Cell death kinetics of AIM2-induced PANoptosis in macrophages lacking executioners

Bone marrow-derived macrophages (BMDMs) derived from wild type (WT) and the indicated executioner knockout mice were infected with *F. novicida* (MOI 100) and cell death analysis was performed using IncuCyte live cell imaging. Real time analysis of cell death kinetics (percent cell death calculated by propidium iodide $(PI)^+$ cells/well) is shown. In some cases, a single WT was used to compare against multiple knockout lines inoculated and stimulated at the same time in the same experiment. The data are plotted separately in the figures for optimal visualization; therefore, the same WT cell death curve appears in multiple panels. Data shown are from one representative experiment with n = 4 technical replicates from a single well per genotype; data are displayed as mean ± SEM. Similar results were obtained in at least 3 independent biological replicate experiments.
